# Comparison of SARS-CoV-2 Variants of Concern Alpha (B.1.1.7) vs. Beta (B.1.351) in Critically Ill Patients: A Multicenter Cohort Study

**DOI:** 10.3389/fmed.2022.828402

**Published:** 2022-03-10

**Authors:** Guillaume Louis, Thibaut Belveyre, Christophe Goetz, Sébastien Gibot, Paul Dunand, Marie Conrad, Rostane Gaci, Sébastien Gette, Nadia Ouamara, Pascale Perez, Cyril Cadoz, Yoann Picard, Nouchan Mellati

**Affiliations:** ^1^Intensive Care Unit, Metz-Thionville Regional Hospital, Mercy Hospital, Metz, France; ^2^Department of Anesthesiology and Intensive Care Medicine, University Hospital of Nancy, Vandoeuvre-Lès-Nancy, France; ^3^Clinical Research Support Unit, Metz-Thionville Regional Hospital, Mercy Hospital, Metz, France; ^4^Medical Intensive Care Unit, University Hospital of Nancy, Nancy, France; ^5^Intensive Care Unit, Metz-Thionville Regional Hospital, Bel Air Hospital, Thionville, France; ^6^Department of Virology, Metz-Thionville Regional Hospital, Mercy Hospital, Metz, France

**Keywords:** COVID-19, intensive care unit, variants, Beta (B.1.351), Alpha (B.1.1.7), clinical indicator

## Abstract

**Objectives:**

The clinical outcomes of the Beta (B.1.351) variant of concern (VOC) of the SARS-CoV-2 virus remain poorly understood. In early 2021, northeastern France experienced an outbreak of Beta that was not observed elsewhere. This outbreak slightly preceded and then overlapped with a second outbreak of the better understood VOC Alpha (B.1.1.7) in the region. This situation allowed us to contemporaneously compare Alpha and Beta in terms of the characteristics, management, and outcomes of critically ill patients.

**Methods:**

A multicenter prospective cohort study was conducted on all consecutive adult patients who had laboratory confirmed SARS CoV-2 infection, underwent variant screening, and were admitted to one of four intensive care units (ICU) for acute respiratory failure between January 9th and May 15th, 2021. Primary outcome was 60-day mortality. Differences between Alpha and Beta in terms of other outcomes, patient variables, management, and vaccination characteristics were also explored by univariate analysis. The factors that associated with 60-day death in Alpha- and Beta-infected patients were examined with logistic regression analysis.

**Results:**

In total, 333 patients (median age, 63 years; 68% male) were enrolled. Of these, 174 and 159 had Alpha and Beta, respectively. The two groups did not differ significantly in terms of 60-day mortality (19 vs. 23%), 28-day mortality (17 vs. 20%), need for mechanical ventilation (60 vs. 61%), mechanical ventilation duration (14 vs. 15 days), other management variables, patient demographic variables, comorbidities, or clinical variables on ICU admission. The vast majority of patients were unvaccinated (94%). The remaining 18 patients had received a partial vaccine course and 2 were fully vaccinated. The vaccinated patients were equally likely to have Alpha and Beta.

**Conclusions:**

Beta did not differ from Alpha in terms of patient characteristics, management, or outcomes in critically ill patients.

**Trial Registration:**

ClinicalTrials.gov, identifier: NCT04906850.

## Introduction

The SARS-CoV-2 virus causes coronavirus disease-2019 (COVID-19), which can result in acute hypoxic respiratory failure secondary to pneumonia among other serious outcomes. It was first detected in Wuhan, China at the end of 2019 and has subsequently caused a quickly spreading and ongoing pandemic that has killed millions of people worldwide (1). Like other RNA viruses, SARS-CoV-2 is highly prone to rapid genetic evolution. Consequently, it became clear by late 2020 that highly transmissible and potentially more virulent variants that might be able to evade natural and vaccine immunity were arising and spreading globally. This alarming threat to public health has led to global efforts to characterize these so-called Variants of Interest (VOIs) (which have potential impacts) and Variants of Concern (VOCs) (which have demonstrated impacts); the aim is to promote global monitoring and research and ultimately inform the ongoing response to the COVID-19 pandemic (2).

On June 22, 2021, the World Health Organization declared that there are four key VOCs, which have been designated Alpha, Beta, Gamma, and Delta. All emerged around late 2020/early 2021. Alpha (PANGO lineage B.1.1.7) was first detected in the United Kingdom while Beta (B.1.351), Gamma (P.1), and Delta (B.1.617.2) were originally from South Africa, Brazil, and India, respectively. The fifth SARS-CoV-2 variant of concern, Omicron (B.1.1.529 lineage), was first publicly announced in South Africa on 25 November 2021 and swept Europe and other regions, becoming rapidly dominant (3). All have multiple mutations in the spike glycoprotein that are thought to increase the transmissibility of the virus. One of these is N501Y, which is present in Alpha, Beta, and Gamma (4). Alpha, Gamma, and Delta all also associate with greater mortality or hospitalization rates than the ancestral strain (5–7). The mutations in Beta have also been suggested to reduce the ability of therapeutic monoclonal antibodies and convalescent serum to neutralize the virus (8, 9). However, almost nothing is known about the mortality rates and other outcomes that associate with this VOC.

In France, the incidence of COVID-19 and its mortality started rising for the third time in January 2021. This coincided with the first confirmed cases of infection with the South African VOC Beta, which arose in a group of individuals who had traveled in December 2020 to Mozambique (which shares a border with South Africa) for a religious gathering (10). This transmission event specifically changed the VOC distribution in the northeast of France. Thus, in early 2021, Alpha accounted for 66% of all new positive polymerase-chain reaction (PCR) test cases in the whole of France while Beta and Gamma together only accounted for 5% (11). By contrast, the prevalence of Beta/Gamma in Moselle (the department in northeast France where our regional hospital is located) at the same time was 41% (12). Beta then spread rapidly in Moselle; by February 2021, epidemiological Flash surveys with precise sequencing showed that only Beta was circulating in this department (13). While Beta has since been superseded by Delta and Omicron both throughout France and our department, the recent predominance of Beta in Moselle has allowed us to assess its outcomes relative to Alpha, the other VOC that quickly started to recirculate in the area during the third wave. Our recent preliminary study on the data of 131 consecutive infected patients suggested that Beta was associated with high short-term mortality and could be more pathogenic than Alpha (14). However, since the sample size of the study was quite limited and it was conducted in a single center, we designed and conducted the VARICOVID study (Evaluation of the New Variant 501Y.V2 of Covid-19). This multicenter study, which is reported here, compared Alpha and Beta in terms of day-60 mortality and other outcomes in critically ill patients who were admitted to four ICUs in January–May 2021.

## Patients and Methods

### Study Design and Ethics

We performed a prospective study with retrospective collection of data in four ICUs, two each in two regional hospitals (CHR Metz-Thionville and CHU Nancy) in the northeast of France. It was approved by the ethics committee of the French Intensive Care Society (n. CE SRLF 21-60), which waived the need for informed consent from the patients due to the retrospective nature of the collection of data. All patients or their relatives were asked by letter whether their anonymized healthcare data could be used for medical research. The VARICOVID study is registered with ClinicalTrials.gov (NCT04906850).

### Patient Selection

Medical chart review was conducted to identify all consecutive adult (≥18 years) patients who (i) had a laboratory-confirmed SARS-CoV-2 infection (as determined by real-time reverse transcriptase-PCR assays of nasal and pharyngeal swabs), (ii) underwent concomitant systematic variant screening, and (iii) were admitted to one of the four ICUs between January 9 and May 15 2021. Patients were admitted based upon the French critical care guideline for patients' admission in a pandemic context (15). The infection was deemed to be Alpha if it had the N501Y mutation and lacked the E484K mutation; Beta if it had both the N501Y and E484K mutations; and non-VOC if it lacked both N501Y and E484K. If lineage information was missing, the strain was classified as “undetermined” (16).

### Primary Outcome, Secondary Outcome, and Other Variables

The variables in the medical charts and electronic reports were recorded by the responsible critical care physicians at each hospital. The primary outcome variable was all-cause mortality 60 days after ICU admission. The secondary outcome variables were: all-cause mortality 28 days after ICU admission; lengths of stay (LOS) in the ICU and hospital; need for and duration of mechanical ventilation (MV) and extracorporeal membrane oxygenation (ECMO); and need for noninvasive ventilation, high flow nasal canula, tracheostomy, vasopressors, or renal replacement therapy.

Other variables were also recorded. They included the following:

- Patient characteristics, namely, patient age, gender, body mass index (BMI), and comorbidities. Overweight was defined as a BMI > 25 kg.m^−2^ while obesity was defined as a BMI > 30 kg.m^−2^. The immunosuppression comorbidity included solid tumors, hematological malignancies, solid organ transplantation, long-term immunosuppressive drugs, or HIV infection (17). Chronic kidney disease was defined as stage 2–5 according to Kidney Disease Improving Global Outcomes guidelines (18).
- Data recorded at the time of ICU admission: ICU center; blood test results; Simplified Acute Physiology Score II (SAPS 2) and Sepsis-Related Organ Failure Assessment (SOFA) scores; delay first symptoms/ICU; the presence of acute kidney injury (AKI); computed tomography (CT) chest impairment; the presence of pulmonary embolism; and whether the patient was pregnant.
- COVID-19 vaccination status, namely, number of vaccinations, the vaccine products used, and dates of administration. This information was obtained from AmeliPro, a centralized COVID-19 vaccine information system in France. Patients were considered fully vaccinated if (i) the second dose of Pfizer-BioNTech BNT162b2 or Moderna mRNA-1273 had been administered at least 14 days before symptom onset/positive PCR test; (ii) the second dose of Oxford-AstraZeneca ChAdOx1 nCoV-19 had been administered at least 21 days before symptom onset/positive PCR test; or (iii) the patient received a first dose of the Oxford vaccine and then Pfizer-BioNTech BNT162b2 or Moderna mRNA-1273 and symptom onset/positive PCR testing occurred at least 14 days after the booster dose with the mRNA vaccine (19, 20). We also determined from the medical records whether the patients had been infected with SARS-CoV-2 previously. These data together allowed us to determine whether the index infection had escaped vaccine- or natural infection-induced immunity.
- Mechanical Ventilation data. All centers followed international guidelines for acute respiratory distress syndrome management, namely, targeting a tidal volume (Vt) of 6 ml/kg of ideal body weight, limited plateau pressure (Pplat) of <30 cmH2O, and prone positioning for severe hypoxemia (21). The following variables of the MV patients were recorded. (i) PaO_2_/FiO_2_ ratio, Vt, maximum Pplat, and positive end-expiratory pressure (PEEP) in the first 24 h. These variables were also used to calculate two additional day 1 variables, namely, the driving pressure (DP, defined as Pplat-PEEP) and the compliance of the respiratory system (CRS, defined as Vt/DP, unit = ml/cmH_2_O). (ii) Delay MV/ICU, need for tracheostomy, development of ventilator-acquired pneumonia during the ICU stay, and day-60 mortality.

### Statistical Analysis

Continuous and categorical variables were expressed as median (interquartile range) and *n* (%), respectively. Patient groups were compared by using the non-parametric Wilcoxon rank-sum test or Fisher's exact test. The probability of survival with Alpha and Beta infections from admission to day 60 was determined by using the Kaplan–Meier estimator followed by log-rank test. Logistic regression analysis was conducted to identify independent risk factors for day-60 mortality in patients with Alpha or Beta. All suspected risk factors were included in the model except for pregnancy and chronic cardiac failure (due to small event numbers) and biological variables apart from lymphocyte counts (due to missing data). The data were expressed as adjusted odds ratios with 95% confidence intervals (CI). The significance level was set at 5%. All analyses were performed by using SAS 9.4 (SAS Inst., Cary, NC).

## Results

During the study period, 365 patients underwent strain testing after admission to one of the four participating ICUs. The virus strain was Alpha in 48% of cases, Beta in 44%, non-VOC in 7%, and undetermined in 1%. In total, 32 patients were excluded from the study because the virus strain was not a VOC (*n* = 26), the virus strain could not be determined (*n* = 5), or the patient refused to participate in the study (*n* = 1). Thus, 333 patients were included in the study.

**Figure 1:**
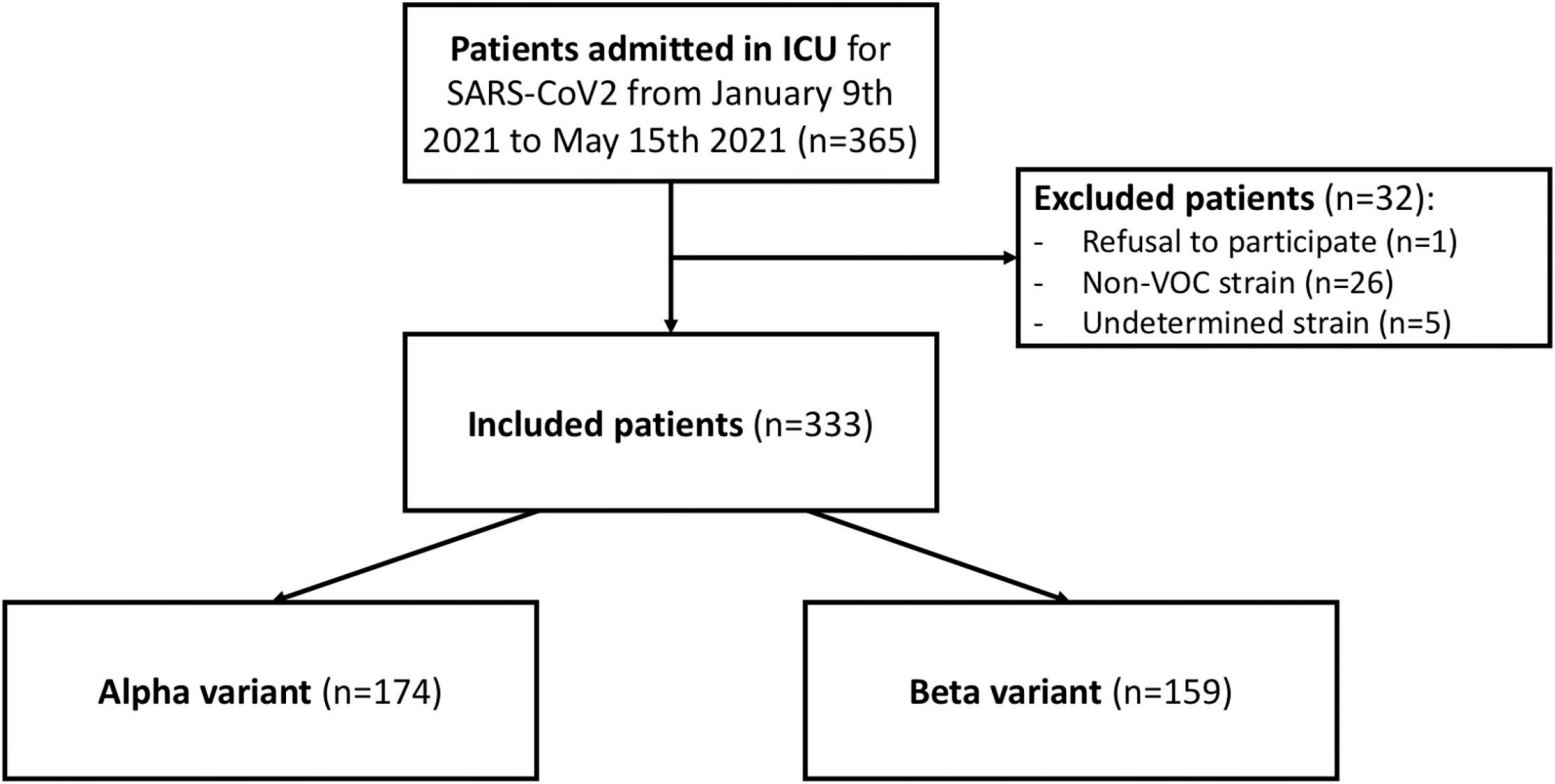
Disposition of the patients in the study.

Of the 333 cases, Alpha accounted for 52% and Beta for 48% of the virus strains (Figure 1). Supplementary Figure 1 shows how these two VOCs evolved over the study period. While Beta was in the majority at the beginning of February, it was gradually supplanted by Alpha.

### Characteristics, Management, and Outcomes of the Total Cohort

**Figure 2:**
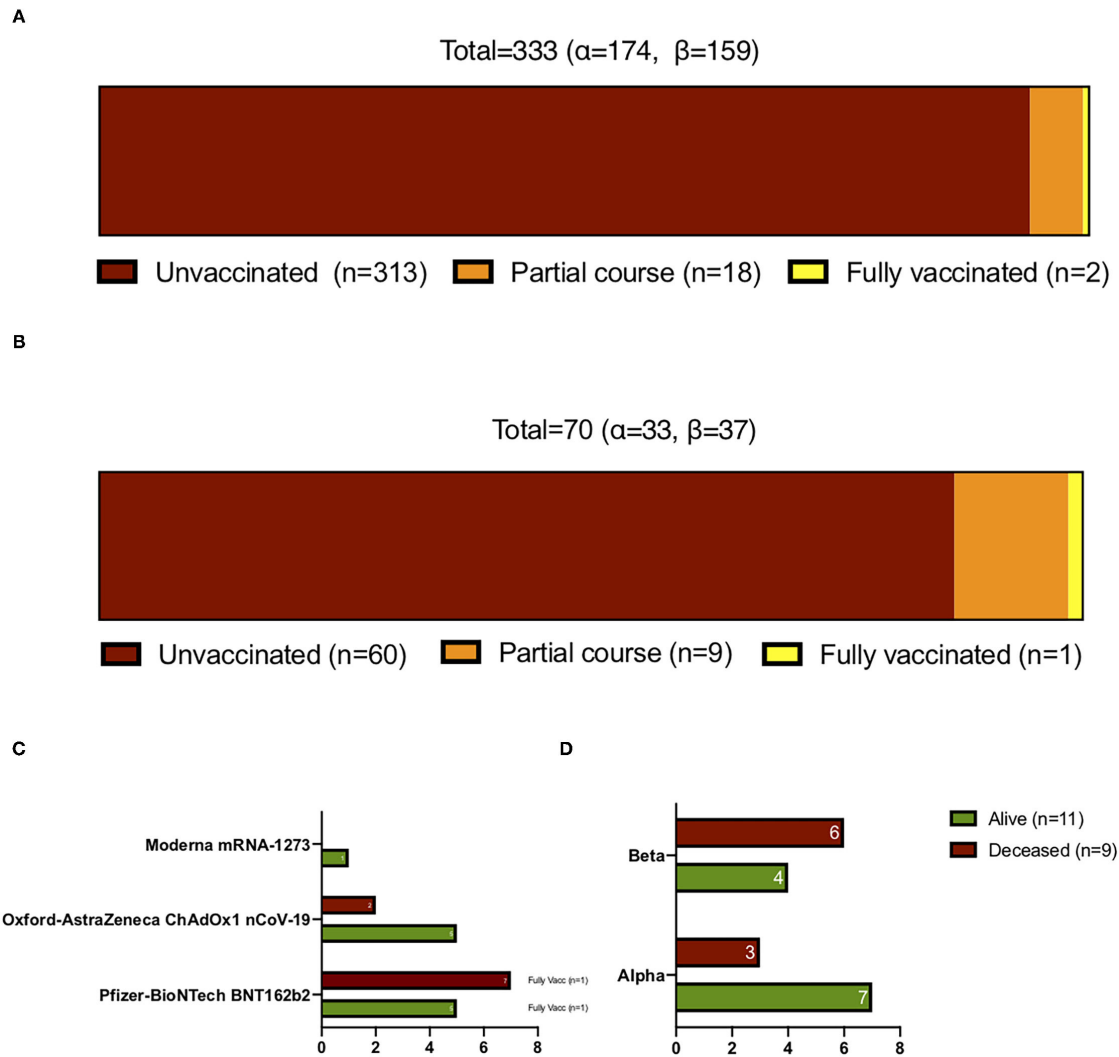
Vaccination status of **(A)** the patients who were admitted to the ICU and **(B,C)** the patients who died by day 60. **(B)** Shows the overall vaccination status of the patients who died while **(C)** shows which vaccines they had received. **(D)** The variant in the vaccinated patients who died by day 60.

The mean time between symptom onset and ICU admission was 9 (6–12) days. Table 1 shows the characteristics of the patients at ICU admission. They had a mean age of 63 (55–69) years, were predominantly male (68%), and 80% had at least one comorbid condition. The most common comorbidities were hypertension, obesity, and diabetes mellitus. The majority of patients were treated in Center 2 (50%); the remaining three ICUs each accounted for 15–18% of the patients. The median SOFA and SAPS 2 scores were 5 (3–7) and 36 (29–45), respectively. Approximately half of the patients had AKI (43%) and a CT chest impairment of ≥50% (56%), 5% had pulmonary embolism, and 2% were pregnant. All patients received glucocorticoids according to RECOVERY protocol (22). No others immunomodulators were used. Only 20 (6%) of the patients had received one or more COVID-19 vaccine doses; most had only received one dose (*n* = 18, 90%) (Figure 2A). The Pfizer-BioNTech vaccine was used for the two fully vaccinated patients and was also the most commonly used vaccine in the partially vaccinated cases (10/18, 56%), followed by the Oxford-Astrazeneca vaccine (7/18, 39%) and Moderna (1/18, 6%) (Figure 2C). Cases of reinfection with the Alpha or Beta variants were not observed.

**Table 1:** Comparison of the Alpha- and Beta-infected patients in terms of their demographic, comorbidity, and characteristics on ICU admission and their primary and secondary outcomes.

**Characteristic**	**All patients** **(*n* = 333)**	**Alpha** **(*n* = 174)**	**Beta** **(*n* = 159)**	***p*** ^*^
Age, years	63 (55–69)	63 (55–68)	63 (55–70)	0.28
Male sex	225 (68)	121 (70)	104 (65)	0.48
BMI, kg/m^2^	30 (27–34)	30 (27–34)	30 (27–35)	0.23
Weight status				0.60
Normal	41 (12)	24 (14)	17 (11)	
Overweight	120 (36)	67 (37)	56 (35)	
Obese	172 (52)	86 (49)	86 (54)	
Hypertension	172 (52)	90 (52)	82 (51)	0.99
Diabetus mellitus	95 (29)	46 (26)	49 (31)	0.40
Cardiovascular disease	50 (15)	24 (14)	26 (16)	0.54
Chronic cardiac failure	8 (2)	5 (4)	3 (2)	0.49
Immunosuppression	25 (8)	17 (10)	8 (5)	0.14
Chronic kidney disease	18 (5)	13 (7)	5 (3)	0.09
COPD	22 (7)	13 (7)	9 (6)	0.66
Center				**0.02**
01	61 (18)	25 (14)	36 (23)	
02	168 (50)	83 (48)	85 (53)	
03	54 (16)	37 (21)	17 (11)	
04	50 (15)	29 (17)	21 (13)	
Platelets, G/L	227 (171–303)	224 (162–286)	229 (180–319)	0.06
D-dimers, μg/L	1465 (909–2191)	1393 (909–2072)	1512 (916–2412)	0.51
PT, %	91 (82–99)	92 (82–100)	89 (81–99)	0.37
Fibrinogen, g/L	7 (6–8)	7 (6–8)	7 (6–8)	0.18
Leukocytes, G/L	10 (7–13)	9 (7–12)	10 (7–13)	0.31
Lymphocytes, G/L	0.64 (0.46–0.98)	0.66 (0.46–1)	0.63 (0.46–0.94)	0.45
SOFA	5 (3–7)	5 (3–7)	4 (3–7)	0.15
SAPS 2	36 (29–45)	36 (29–46)	36 (29–42)	0.24
Delay 1^st^ symptoms/ICU	9 (6–12)	10 (6–12)	9 (6–12)	0.24
AKI	144 (43)	69 (40)	75 (47)	0.18
CT chest impairment				0.78
<10%	6 (2)	4 (2)	2 (1)	
10–25%	30 (9)	15 (9)	15 (10)	
25–50%	98 (29)	50 (30)	48 (32)	
50–75%	135 (41)	76 (45)	59 (39)	
>75%	50 (15)	24 (14)	26 (17)	
Pulmonary embolism	17 (5)	10 (6)	7 (4)	0.63
Pregnancy	5 (2)	2 (1)	3 (2)	0.68
All-cause day-60 mortality	70 (21)	33 (19)	37 (23)	0.35
All-cause day-28 mortality	62 (19)	30 (17)	32 (20)	0.57
ICU LOS, days	11 (5–23)	11 (5–23)	10 (6–23)	0.68
Hospital LOS, days	16 (10–29)	16 (10–30)	16 (11–29)	0.98
Need for MV	203 (61)	105 (60)	98 (61)	0.82
Duration of MV, days	15 (8–26)	14 (8–26)	15 (7–26)	0.89
Need for ECMO	14 (4)	4 (2)	10 (6)	0.10
Duration of ECMO, days	9 (5–17)	8 (5–9)	13 (5–18)	0.36
Need for HFNC	266 (80)	141 (81)	125 (79)	0.59
Need for NIV	146 (44)	79 (45)	67 (42)	0.58
Need for tracheostomy	22 (7)	14 (8)	8 (5)	0.28
Need for vasopressors	165 (50)	87 (50)	78 (49)	0.83
Need for RRT	25 (8)	17 (10)	8 (5)	0.14

*The data are expressed as median (interquartile range) or n (%)*.

* *p-values were obtained by comparing Alpha- and Beta-infected patients with the non-parametric Wilcoxon rank-sum test or Fisher's exact test. AKI, acute kidney injury; BMI, body mass index; CT, computed tomography; COPD, chronic obstructive pulmonary disease; Delay 1st symptoms/ICU, delay between first symptoms and ICU admission; ECMO, extracorporeal membrane oxygenation; HFNC, high flow nasal canula; ICU, intensive care unit; LOS, length of stay; MV, mechanical ventilation; NIV, noninvasive ventilation; PT, prothrombin time; RRT, renal replacement therapy; SAPS2, Simplified Acute Physiology Score 2; SOFA, Sepsis-related Organ Failure Assessment*.

*The bold values indicate the difference is significant (p <0.05)*.

Mortality rates at day 60 and day 28, were 21 and 19% respectively (Table 1). Sixty-one of the 313 unvaccinated, one of the two fully vaccinated patients, and eight of the 18 partially vaccinated patients died by day 60 (Figure 2B). The demographics, comorbidities, management, and outcomes of the partially and fully vaccinated patients are presented in Supplementary Material.

The patients spent a median of 11 and 16 days in the ICU and hospital, respectively. MV and ECMO were required in 61 and 4% of cases for median durations of 15 and 9 days, respectively. Most patients required high flow nasal cannulas (80%), half required noninvasive ventilation (44%) and vasopressors (50%), and tracheostomy and renal replacement therapy were conducted in 7 and 8%, respectively (Table 1).

The characteristics of the 203 patients who underwent MV are presented in Table 2. The median PaO_2_/FiO_2_ ratio was 87 mmHg, which indicates severe hypoxemia. Median maximum PPlat, Vt, and PEEP on day 1 were 27 cmH_2_O, 410 ml, and 12 cmH_2_O, respectively. Median DP and CRS were 14 and 27 ml/cmH_2_O, respectively. 11 % of patients required tracheostomy, 52% developed ventilator-acquired pneumonia, and 29% died by day 60.

**Table 2:** Comparison of the 203 Alpha- and Beta-infected patients who underwent mechanical ventilation in terms of mechanical ventilation characteristics and day-60 mortality.

**Characteristic**	**All patients** **(*n* = 203)**	**Alpha** **(*n* = 105)**	**Beta** **(*n*=98)**	***p*** ^*^
MV duration	15 (8–26)	14 (8–26)	15 (7–26)	0.89
Day 1 PaO_2_/FiO_2_	87 (63–108)	87 (64–115)	84 (62–101)	0.16
Day 1 max PPlat (cmH_2_O)	27 (24–29)	26 (24–28)	27 (24–30)	0.06
Day 1 tidal volume (mL)	410 (360–450)	420 (360–450)	405 (360–450)	0.80
Day 1 PEEP (cmH_2_O)	12 (10–14)	12 (10–14)	12 (10–14)	0.48
DP (cmH_2_0)	14 (12–17)	14 (12–17)	15 (13–18)	0.45
CRS (ml/cmH_2_O)	27 (22–35)	28 (22–35)	26 (22–34)	0.35
Delay MV/ICU	0 (0–1)	0 (0–2)	0 (0–1)	0.20
Need for tracheostomy	22 (11)	14 (13)	8 (8)	0.27
VAP	106 (52)	52 (50)	54 (55)	0.48
Day-60 mortality	59 (29)	29 (28)	30 (31)	0.65

*The data are expressed as median (interquartile range) or n (%)*.

* *p-values were obtained by comparing Alpha- and Beta-infected patients with the non-parametric Wilcoxon rank-sum test or Fisher's exact test*.

*CRS, compliance of the respiratory system; Delay MV/ICU, delay between admission in ICU and start of mechanical ventilation; DP, driving pressure; ICU, intensive care unit; max PPlat, maximum plateau pressure; MV, mechanical ventilation; PEEP, positive end-expiratory pressure; VAP, ventilator-acquired pneumonia*.

### Comparison of Patients With Alpha and Beta in Terms of ICU Variables, Outcomes, and Vaccination Status

**Figure 3:**
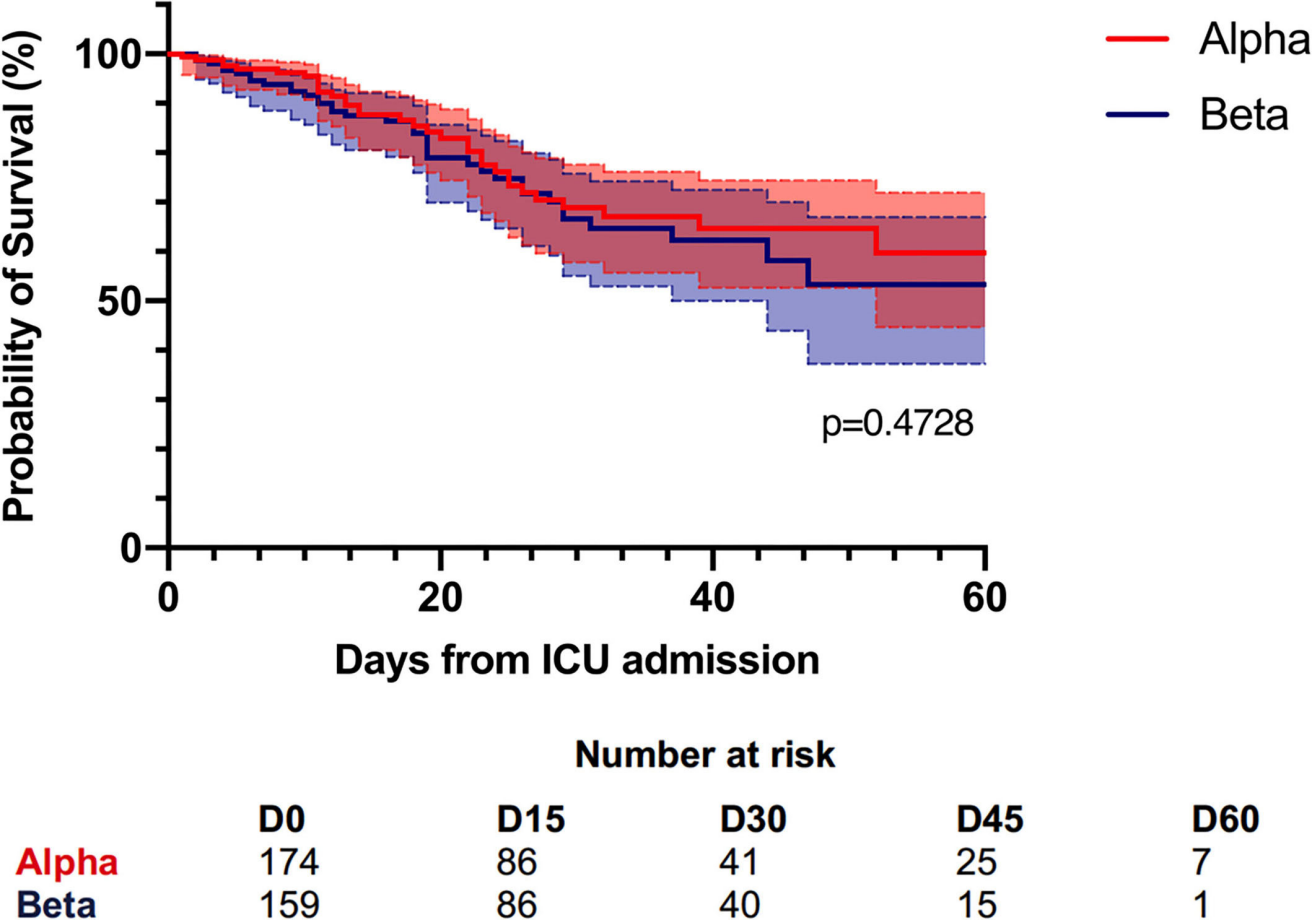
Kaplan-Meier survival curves of the patients who were admitted to an ICU for an infection with Alpha or Beta (*n*=333).

On univariate analyses, the Alpha- and Beta-infected patients did not differ significantly in terms of any variables except center (*p* = 0.02). The day-60 mortality rates of the two groups were 19 and 23%, respectively (Table 1). Kaplan-Meier analyses did not find a significant difference between the two groups in terms of probability of dying (*p* = 0.47) (Figure 3). The Alpha- and Beta-infected patients on MV also did not differ in terms of any variables, including day-60 mortality (Table 2). The Alpha- and Beta-infected patients underwent very similar treatment regimens (Tables 1, 2).

Like the ICU patients as a whole, the vaccinated patients were equally likely to have Alpha and Beta (10 had Alpha, 10 had Beta) (Figure 2D). The vaccinated patients with Beta seemed somewhat to be more likely to die by day 60 than the vaccinated patients with Alpha (60 vs. 30%) (Figure 2D).

### Factors That Associated With Day-60 Mortality in the Alpha- and Beta-Infected Patients

The Alpha- and Beta-infected groups were each divided into two subgroups depending on mortality at day 60. On univariate analysis, the Alpha-infected patients who died by day 60 were significantly older (68 vs. 62 years, *p* = 0.002), had a higher SOFA score on admission to the ICU (7 vs. 5, *p* = 0.001), and were more likely to have hypertension (76 vs. 48%, *p* = 0.01) or diabetes mellitus (45 vs. 23%, *p* = 0.04) than the Alpha-infected patients who were alive by day 60. On multivariate analysis, only SOFA score (OR = 1.34, 95%CI = 1.11–1.63, *p* = 0.003) remained a significant factor that predicted day-60 mortality in Alpha-infected patients (Table 3A).

**Table 3:** Logistic regression analysis to determine the factors that associated with mortality 60 days after admission in ICU for an infection with (A) Alpha (*n* = 161^a^) or (B) Beta (*n* = 139^b^).

**A. Characteristics**	**Deceased at day 60 (*n* = 29)**	**Alive at day 60 (*n*=132)**	***p*** ^*^	OR (95%CI)^**^	***p*** ^**^
Age, years	68 (62–71)	62 (53–68)	**0.002**	1.06 (0.99–1.14)	0.12
Female sex	8 (28)	40 (30)	0.83	0.65 (0.21–2.03)	0.46
Lymphocytes, G/L	0.67 (0.46–0.88)	0.65 (0.47–1.02)	0.73	0.74 (0.27–2.02)	0.56
SOFA score	7 (5–8)	5 (3–7)	**0.001**	1.34 (1.11–1.63)	**0.003**
Delay 1st symptoms/ICU	7 (5–11)	9 (7–12)	0.20	0.94 (0.85–1.04)	0.20
Center			0.19		
01	7 (24)	17 (13)		3.4 (0.82–14.17)	0.09
02	14 (48)	57 (43)		Ref.	
03	6 (21)	31 (23)		1.37 (0.33–5.7)	0.49
04	2 (7)	27 (20)		0.18 (0.03–1.06)	0.06
Weight status			0.61		
Normal	4 (14)	19 (14)		Ref.	
Overweight	13 (45)	46 (35)		0.77 (0.16–3.68)	0.30
Obese	12 (41)	67 (51)		2.2 (0.49–9.85)	0.74
Hypertension	22 (76)	64 (48)	**0.01**	2.01 (0.61–6.7)	0.25
Diabetus mellitus	13 (45)	31 (23)	**0.04**	2.83 (0.92–8.69)	0.07
Cardiovascular disease	6 (21)	17 (13)	0.38	1.14 (0.3–4.31)	0.85
Immunosuppression	4 (14)	11 (8)	0.48	0.82 (0.17–4.03)	0.81
Chronic kidney failure	3 (10)	8 (6)	0.42	0.83 (0.13–5.3)	0.84
COPD	4 (14)	8 (6)	0.23	4.46 (0.78–25.54)	0.09
**B. Characteristics**	**Deceased at Day 60 (*n* = 31)**	**Alive at Day 60 (*n* = 108)**	***p*** ^*^	OR (95%CI)^**^	***p*** ^**^
Age, years	68 (65–73)	62 (53–68)	**0.001**	1.1 (1.03–1.18)	**0.01**
Female sex	11 (35)	39 (36)	0.99	1.2 (0.38–3.77)	0.75
Lymphocyte, G/L	0.56 (0.44–0.94)	0.65 (0.47–0.95)	0.39	0.88 (0.29–2.64)	0.82
SOFA score	6 (4–7)	4 (3–6)	**0.03**	1.22 (1.02–1.48)	**0.03**
Delay 1st symptoms /ICU	8 (4–11)	9 (6–12)	0.44	1.01 (0.91–1.12)	0.90
Center			0.49		
01	8 (26)	25 (23)		0.58 (0.15–2.32)	0.44
02	18 (58)	51 (47)		Ref.	
03	3 (10)	14 (13)		0.78 (0.15–4.14)	0.77
04	2 (6)	18 (17)		0.11 (0.02–0.66)	**0.02**
Weight status			0.51		
Normal	5 (16)	10 (9)		Ref.	
Overweight	11 (35)	40 (37)		0.94 (0.16–5.52)	0.95
Obese	15 (48)	58 (54)		1.56 (0.28–8.79)	0.62
Hypertension	21 (68)	51 (47)	0.07	1.28 (0.4–4.1)	0.68
Diabetus mellitus	11 (35)	34 (31)	0.67	0.52 (0.15–1.79)	0.30
Cardiovascular disease	11 (35)	13 (12)	**0.01**	4.03 (1.02–15.89)	**0.046**
Immunosuppression	3 (10)	4 (4)	0.19	4.19 (0.63–27.96)	0.14
Chronic kidney failure	4 (13)	1 (1)	**0.01**	10.29 (0.64–165)	0.10
COPD	2 (6)	6 (6)	0.99	0.58 (0.07–4.64)	0.61

a *13 patients were excluded from the analysis because of missing lymphocyte data*.

b *20 patients were excluded from the analysis because of missing lymphocyte and delay between symptoms and ICU admission data*.

* *Wilcoxon rank-sum test or Fisher's exact test*.

** *Logistic regression*.

*COPD, Chronic Obstructive Pulmonary Disease; SOFA, Sepsis-related Organ Failure Assessment*.

*The bold values indicate the difference is significant (p <0.05)*.

Like the Alpha-infected group, the patients in the Beta-infected group who died by day 60 were significantly older (68 vs. 62 years, *p* = 0.001) and had a higher SOFA score on admission to the ICU (6 vs. 4, *p* = 0.03) than the Beta-infected patients who were alive at day 60. Unlike the Alpha group, they were more likely to have cardiovascular disease (35 vs. 12%, *p* = 0.01) or chronic kidney failure (13 vs. 1%, *p* = 0.01) on univariate analysis. On multivariate analysis, age (OR = 1.1, 1.03–1.18, *p* = 0.01), SOFA score (OR = 1.22, 1.02–1.48, *p* = 0.03), and cardiovascular disease (OR = 4.03, 1.02–15.89, *p* = 0.046) remained factors that significantly predicted day-60 mortality in Beta-infected patients. Center also appeared as a significant factor in the Beta-infected patients: Center 4 associated with significantly less mortality (OR = 0.11, 0.02–0.66, *p* = 0.02) (Table 3B).

## Discussion

### Alpha and Beta Do Not Differ in Mortality Rates

This cohort study showed that Beta-infected patients did not differ from Alpha-infected patients in terms of their baseline characteristics, management, and outcomes. Specifically, they did not differ in terms of day-60 mortality (19 and 23%, respectively; *p* = 0.35) and day-28 mortality (17 and 20%, respectively; *p* = 0.57). These mortality rates are similar to the mortality rates that associate with the wild-type strain: for example, two multicenter cohort studies showed that the 28-day mortality of critically ill French patients infected with the wild-type strain during the first COVID-19 wave ranged from 18 to 31% (23, 24). Thus, Alpha and Beta appear to have similar mortality rates in critically ill patients as the wild-type strain. This has been observed previously for Alpha in a retrospective single-center German cohort study on 160 critically ill patients (25). However, little is known about the relative mortality of Beta in ICU patients: to our knowledge, the only study on this to date is our recent preliminary single-center study, which reported that Beta associated with a 60-day mortality rate of 30% in ICU patients (14). This paucity of research on Beta may reflect its relative rarity. For example, a large European study showed that Alpha, Beta, and Gamma associated with significantly higher odds of hospitalization and ICU admission compared to non-VOC strains (26). However, Alpha was by far the most frequently reported VOC (966 hospitalized cases and 121 ICU cases) in this study; there were far fewer Beta cases (74 hospitalized cases and 9 ICU cases). A recent study suggests that Beta associates with increased hospital mortality in South African patients (relative to the first wave). However, sequencing has not been done routinely (6). A recent meta-analysis suggests an increased mortality of Beta variant compared to wild type strain. Plus, in this meta-analysis, the Beta and Delta variants were described as risker than the Alpha and Gamma variants (27). Nevertheless, none of the studies cited in the meta-analysis were specific to critically ill patient or compared variants with each other. Plus, the Beta variant proportion was quite low compared to other variants in these studies (Alpha particularly) which can lead to a bias.

### SOFA Score, Center, Age, and Comorbidities Associate With Mortality in Patients With Alpha and Beta

On multivariate analysis, SOFA score predicted day-60 mortality in both Alpha- and Beta-infected patients. The association with SOFA score and COVID-19 mortality has been observed previously (28). Nevertheless, recent findings tend to show that SOFA score possesses inadequate discriminant accuracy to be used for ventilated COVID-19 patient. It could explain why the SOFA score described in our study is low comparing to mortality (29). With regard to center, Center 4 associated with lower Beta-related mortality rates compared to the reference center (Center 2). The association with Center 4 may reflect the fact that of the four ICUs, this ICU was the less impacted by patient numbers relative to bed capacity than the reference center during the third COVID-19 wave. Such associations have been observed previously. For example, a meta-analysis showed that ICU capacity strain associated with increased mortality in nine of 12 cohort studies that were published between 1999 and 2015 (i.e., before COVID-19 emerged) (30). This has also been observed during the COVID-19 pandemic: a cohort study of 8,516 patients who were admitted to 88 US Veterans Affairs hospitals showed that strain on critical care capacity associated with increased COVID-19 ICU mortality (31). We also observed that age was a predictive factor for Beta-related mortality on multivariate analysis and for Alpha-related mortality on univariate analysis. This is generally consistent with previous findings: age is a well-known predictor of COVID-19 outcomes (32). Finally, we found that Beta-related mortality associated with cardiovascular disease on multivariate analysis and diabetes mellitus on univariate analysis, and that Alpha-related mortality associated with cardiovascular and chronic kidney disease on univariate analyses. These findings have been noted previously (24, 33).

### Vaccination Is Protective

As expected, the vast majority of the study patients (94%) had not been vaccinated against COVID-19. This contrasts with the single and double vaccination rates in France during the January–May 2021 study period, which respectively rose in an exponential fashion from 3 and 0.1% in 1 February 2021 to 48 and 28% in 21 June 2021 (34). Moreover, most of the vaccinated ICU patients (90%) had only received one dose. These observations support the strong existing evidence showing that emergency care/hospitalization due to breakthrough COVID-19 is an exceedingly rare event in fully vaccinated patients (35). This is partially supported by a growing body of evidence showing that the ability of Alpha to escape from vaccine-induced immunity is limited and vaccines remain clinically effective against this variant (36, 37). This also appears to be true for Beta, at least with respect to the Pfizer-BioNTech vaccine: a study in Qatar that was conducted when Alpha and Beta were circulating at similar levels showed that while two doses of the Pfizer-BioNTech vaccine were more effective at preventing Alpha infection than Beta infection (89.5 vs. 75%), they provided high protection from severe, critical, or fatal COVID-19 regardless of the strain (97.4%) (38). It should be noted that this may not be true for the Oxford-AstraZeneca vaccine: a clinical trial showed recently that this vaccine had poor efficacy against Beta infection (39). The vaccinated patients were equally like to be infected with Alpha and Beta, like the ICU patients as a whole, which suggests that the immune status of these mostly singly-vaccinated patients was equally ineffective with both VOCs. Beta seemed to be fatal more often in the vaccinated patients than Alpha (60 vs. 30%) but the numbers are too small to determine whether this trend is real.

The patients were more likely to be vaccinated with the mRNA vaccines than the Oxford-Astrazeneca vaccine (65 vs. 35%). This reflects the fact that the High Authority of Health recommended that northeast France should have access to RNA vaccines during the study period (40).

Our study also showed that there were no cases of severe reinfection with Alpha or Beta admitted in ICU. This is supported by previous studies that show patients who have had a wild-type infection subsequently acquire Alpha or Beta infections only very rarely (41). Moreover, such reinfections generally associate with non-severe illness (42). However, it should be noted that because the serological status of each patient before their admission into the ICU was not known, we could not formally prove the absence of reinfection.

### Study Strengths and Limitations

To date, this is one of the largest studies to compare the characteristics and outcomes of critically ill patients who were infected with either of two simultaneously circulating VOCs and who underwent similar treatment regimens in the same settings. To our knowledge, it is also the largest study on Beta. Moreover, the study also has a multicenter design since it focused on four highly impacted ICUs.

Study limitations include its retrospective design concerning collection of data. Moreover, only four ICUs in two regional hospitals participated because Beta evinced only a very modest presence in the rest of the country. Another study limitation was that we did not examine the role of viral load, which has been shown to predict disease severity (43). This reflects the fact that we did not have these data. In addition, we defined Beta on the basis of VOC screening as having the N501Y and E484K mutations. Since Beta and Gamma have both of these mutations, it remains possible that a few of the Beta specimens were actually Gamma specimens. However, this is quite unlikely given that epidemiological Flash surveys with precise sequencing showed that only Beta was circulating in the region during the study period (12).

The two VOCs of interest in our study (Alpha and Beta) have now been supplanted regionally and globally by the new VOC designated Delta and Omicron. Nonetheless, our study remains of interest because very few clinical data have been published on Beta to date. Our findings will also help in the case that this variant re-emerges in the future.

## Conclusions

The Alpha and Beta VOCs did not differ in terms of ICU patient characteristics, management, and outcomes, including day-60 mortality. Better understanding of these variants including ongoing and future ones is essential. Thereby, it is vital that we remain vigilant, monitor and evaluate the variants arising around the globe.

## Data Availability Statement

The original contributions presented in the study are included in the article/Supplementary Material, further inquiries can be directed to the corresponding author.

## Ethics Statement

The studies involving human participants were reviewed and approved by Ethics Committee of the French Intensive Care Society (n. CE SRLF 21-60). Written informed consent for participation was not required for this study in accordance with the national legislation and the institutional requirements.

## Author Contributions

GL, TB, SGi, NM, and YP: lead investigators. GL: study conception and design and protocol development. CG and NO: trial methodologist and statistical analysis. GL, TB, SGi, SGe, MC, PD, YP, NM, CC, PP, and RG: data collection. GL, TB, and CG: data curation and interpretation. GL and TB: critical revision and approval for submission. All authors agree to be both personally accountable for their own contributions and to ensure that questions related to the accuracy or integrity of any part of the work, even ones in which the author was not personally involved, are appropriately investigated, resolved, the resolution documented in the literature, and read and approved the final manuscript.

## Conflict of Interest

GL has received financial support from Pfizer and Fresenius to participate in scientific meetings during the 36 months prior to publication. The remaining authors declare that the research was conducted in the absence of any commercial or financial relationships that could be construed as a potential conflict of interest.

## Publisher's Note

All claims expressed in this article are solely those of the authors and do not necessarily represent those of their affiliated organizations, or those of the publisher, the editors and the reviewers. Any product that may be evaluated in this article, or claim that may be made by its manufacturer, is not guaranteed or endorsed by the publisher.
