# Supplementary material for: Comparison of SARS-CoV-2 Variants of Concern Alpha (B.1.1.7) vs. Beta (B.1.351) in Critically Ill Patients: A Multicenter Cohort Study
Source: Front Med (Lausanne). 2022 Mar 10;9:828402. doi: 10.3389/fmed.2022.828402 (PMC8960192; doi:10.3389/fmed.2022.828402)
Supplement: Supplementary file 1 [file Data_Sheet_1.docx]

**Electronic Supplementary Appendix**

**Comparison of SARS-CoV-2 variants of concern Alpha (B.1.1.7) *versus* Beta (B.1.351) in critically ill patients: a multicenter cohort study**

By Louis *et al.*

**Supplementary Figure 1:**

**Evolution of the Alpha and Beta variants over the study period.**

**ESM Table 1: Demographic characteristics, comorbidities, and outcomes of the 20 partially and fully vaccinated SARS-CoV2 positive patients in the study cohort**

| **Sex** | **Age** | **Variant** | **BMI, kg/m^2^** | **Hypertension** | **Diabetus mellitus** | **CV disease** | **Immunosuppression** | **Chronic kidney disease** | **COPD** | **Vaccine N°1** | **Vaccine N°2** | **Delay 1^st^ injection /ICU** | **MV** | **MV duration, days** | **SAPS2** | **ICU LOS, days** | **D28 outcome** | **D60 outcome** |
| --- | --- | --- | --- | --- | --- | --- | --- | --- | --- | --- | --- | --- | --- | --- | --- | --- | --- | --- |
| F | 55 | Alpha | 26.1 | Yes | Yes | Yes | No | Yes | No | PB | PB | 58 | No |  | 57 | 7 | D | D |
| M | 65 | Beta | 25.0 | No | No | No | No | No | No | AZ | Not done | 12 | Yes | 4 | 61 | 4 | D | D |
| M | 75 | Beta | 24.9 | Yes | Yes | Yes | No | Yes | No | PB | Not done | 15 | No | 10 | 66 | 9 | D | D |
| F | 68 | Beta | 28.0 | Yes | No | No | Yes | No | No | AZ | Not done | 25 | Yes | 4 | 57 | 19 | D | D |
| M | 68 | Beta | 51.5 | Yes | No | Yes | No | No | Yes | PB | Not done | 18 | Yes | 27 | 35 | 31 | A | D |
| M | 71 | Alpha | 25.9 | Yes | No | No | Yes | No | No | PB | Not done | 15 | Yes | 19 | 49 | 19 | D | D |
| M | 72 | Beta | 21.6 | No | No | No | Yes | No | No | PB | Not done | 14 | No |  | 86 | 2 | D | D |
| M | 72 | Beta | 27.8 | No | No | No | No | No | Yes | PB | Not done | 23 | Yes | 10 | 40 | 9 | D | D |
| M | 70 | Alpha | 26.3 | No | Yes | No | No | No | No | PB | Not done | 14 | Yes | 29 | 35 | 28 | A | D |
| M | 61 | Alpha | 39.3 | Yes | Yes | No | No | No | No | AZ | Not done | 16 | No |  | 34 | 4 | A | A |
| F | 46 | Beta | 32.5 | Yes | Yes | No | No | No | No | PB | Not done | 31 | No |  | 20 | 7 | A | A |
| F | 51 | Alpha | 51.9 | No | Yes | Yes | No | Yes | No | AZ | Not done | 60 | No |  | 31 | 3 | A | A |
| F | 60 | Beta | 38.9 | No | No | No | No | No | No | PB | Not done | 25 | No |  | 36 | 16 | A | A |
| F | 61 | Beta | 34.3 | No | No | No | No | No | No | Mo | Not done | 6 | No |  | 20 | 3 | A | A |
| M | 69 | Alpha | 30.0 | No | No | No | No | No | No | AZ | Not done | 13 | No |  | 29 | 9 | A | A |
| M | 55 | Alpha | 22.4 | No | No | No | Yes | Yes | No | PB | Not done | 32 | No |  | 34 | 30 | A | A |
| M | 62 | Alpha | 24.6 | Yes | Yes | No | No | No | No | AZ | Not done | 18 | Yes | 4 | 29 | 10 | A | A |
| M | 63 | Beta | 28.4 | Yes | No | No | Yes | No | No | PB | PB | 42 | No |  | 29 | 6 | A | A |
| M | 59 | Alpha | 35.6 | Yes | Yes | No | No | No | No | PB | Not done | 4 | Yes | 12 | 23 | 17 | A | A |
| M | 67 | Alpha | 26.0 | No | Yes | No | Yes | No | No | AZ | Not done | 16 | No |  | 44 | 8 | A | A |
| Male  =14 | Mean = 63.5 | Alpha= 10 /Beta=10 | Mean=  31.5 | Yes=10 | Yes=9 | Yes=4 | Yes=6 | Yes=4 | Yes=2 |  |  | Mean= 22.9 | Yes=9 | Mean=  13.2 | Mean=40.8 | Mean=  12.1 | D=8 | D=9 |

Abbreviations: AZ: AstraZeneca; BMI: body mass index; CV: cardiovascular; COPD: chronic obstructive pulmonary disease; F: female; ICU: intensive care unit; LOS: length of stay; M: male; Mo: Moderna; MV: mechanical ventilation; SAPS: Simplified Acute Physiology Score; PB: Pfizer/BioNTech.
